# Therapeutic Potential of Umbilical Cord Mesenchymal Stromal Cells Transplantation for Cerebral Palsy: A Case Report

**DOI:** 10.1155/2013/146347

**Published:** 2013-03-03

**Authors:** Liming Wang, Haijie Ji, Jianjun Zhou, Jiang Xie, Zhanqiang Zhong, Ming Li, Wen Bai, Na Li, Zijia Zhang, Xuejun Wang, Delin Zhu, Yongjun Liu, Mingyuan Wu

**Affiliations:** ^1^Cell Therapy Center, 323 Hospital of People's Liberation Army, Xi'an 710054, China; ^2^Alliancells Institute of Stem Cells and Translational Regenerative Medicine & Alliancells Bioscience Co., Ltd., Tianjin 300381, China; ^3^Zhongyuan Union Stem Cell Bioengineering Stock Company, Tianjin 300050, China; ^4^Beijing Alliancells-PuRui Bioscience Co., Ltd., Beijing 100052, China; ^5^Department of Cell Biology, Oklahoma State University, Tulsa, OK 74106, USA; ^6^Harold Hamm Oklahoma Diabetes Center and Section of Endocrinology and Diabetes in the Department of Internal Medicine, University of Oklahoma Health Sciences Center, Oklahoma City, OK 73104, USA

## Abstract

Cerebral palsy is the most common motor disability in childhood. In current paper, we first report our clinical data regarding administration of umbilical cord mesenchymal stem cells (MSCs) transplantation in treatment of cerebral palsy. A 5-year-old girl with cerebral palsy was treated with multiple times of intravenous and intrathecal administration of MSCs derived from her young sister and was followed up for 28 months. The gross motor dysfunction was improved. Other benefits included enhanced immunity, increased physical strength, and adjusted speech and comprehension. Temporary low-grade fever was the only side effect during the treatment. MSCs may be a safe and effective therapy to improve symptoms in children with cerebral palsy.

## 1. Introduction

Cerebral palsy is a group of severe disorders in the development of movement and posture occurred in developing fetal or infant brain, often accompanied with disturbances of sensation, cognition, communication, perception, and/or behavior and/or by a seizure [1]. The causes of cerebral palsy are heterogeneous with no single etiology predominating, and the main etiological factors are periventricular leukomalacia, intrapartum asphyxia, cerebral dysgenesis, and intracranial hemorrhage [2]. The prevalence in children aged 3–10 years is 2–4 per 1000 [3]. The essential feature of children with cerebral palsy is an early-onset neuromotor impairment resulting from a nonprogressive pathology in immature brain [4]. Roughly half of children with cerebral palsy also have symptoms of nonneuromotor impairments, such as cognitive disabilities, epilepsy, speech and language difficulties, primary sensory impairment, and behavioral challenges [5].

Conventional therapies for cerebral palsy include physical and occupational therapy, oral medications, and orthopedic surgery for supportive and rehabilitative approaches [6]. Stem cell therapy is considered as a novel approach in the treatment of cerebral palsy via replacing injured or dead neuronal cells and has proven effective in restoring injured organs and tissues in animal models [7]. Mesenchymal stem cells (MSCs) were first identified in 1976 in the stromal compartment of bone marrow [8] and are currently referred to also as mesenchymal stromal cells [9]. Previously, a few case reports showed the positive clinical benefits of mesenchymal stromal cells in the treatment of neurological diseases including spinal cord injury and basilar artery dissection [10–12]. Recently, a case report that intrathecal infusion of autologous bone marrow mononuclear cells in a cerebral palsy patient suggested the cell transplantation is effective and safe with encouraging functional outcome improvements [13].

Here we present a pediatric case to determine whether combined intrathecal and intravenous administration of umbilical cord MSCs is safe and effective in a patient with cerebral palsy.

## 2. Case Report

The study was approved by the Institutional Review Boards of participating institutions at 323 Hospital of PLA (Xi'an, China) and was conducted according to the principles of the Declaration of Helsinki. Ethical consents for MSCs transplantation were obtained from the patient and her parents.

A 5-year-old girl suffering from cerebral palsy was referred to our hospital in December 2008. The major symptom was congenital growth retardation of motor function including ambulation with tumble, disability of standing up by her, and severe dysarthria precluding speech. Physical examinations indicated that muscular tension of limbs was normal, but fine motor skills were poor. Meanwhile, her apprehension and thinking were worse than those of other same-age children. Her functional independence measure was 48/126. She had no epilepsia and chorea history. She showed limited response for other treatments including neurotrophic drugs and rehabilitation training.

MSCs were derived from umbilical cord of her younger sister and prepared as described previously with some modifications [14]. Briefly, the whole process of MSCs preparation was performed in a good manufacturing practice (GMP) facility in Alliancells Institute of Stem Cells and Translational Regenerative Medicine located in Tianjin, China. The umbilical cord was minced into 1-2 mm^3^ fragments and incubated with 0.075% collagenase type II for 30 min and then 0.125% trypsin for another 30 min with gentle agitation at 37°C. The digested mixture was passed through a 100 *μ*m filter to obtain cell suspension, and then the MSCs were expanded by *in vitro* culturing. The releasing criteria included cell viability (>95%), free from bacterial and viral contamination, absence of endotoxin and immunophenotyping showing expression of CD73, CD90, and CD105 (>90%), and absence of CD34, CD45, CD14, CD19, and HLA DR (<2%).

For each treatment, a total of 5–10 × 10^6^ MSCs in 20 mL solution were administered, of which 18 mL were delivered intravenously and 2 mL by subarachnoid injection via lumbar puncture. A total of seven treatments were processed from December 2008 to June 2009 (Table 1). During the treatment period, the patient had one episode of temporary fever without needing an additional treatment. No other medical treatment except rehabilitation training was performed.

 The patient was followed up for 28 months since the last transplantation of MSCs. Symptoms before and after MSCs treatment were carefully compared (Table 2). The major improvement was the reduction of ambulation with tumble. The patient was able to stand up by herself. Other improvements included enhanced immunity, increased physical strength, and adjusted speech and comprehension which also were observed. Multiple times of examinations including chest X-rays, routine blood test, and liver and renal function test showed normal parameters.

## 3. Discussion

Previous clinical trials showed that subarachnoid placement of stem cells was safe without long-term side effects [15]. In current paper, temporary low-grade fever after administration of umbilical cord MSCs was observed and resolved without any treatment within 24 h. At the time of writing, no major side effects are observed during the followup for 28 months.

The MSCs contribute to substantial neuroprotection and neuroregeneration in the brain [16, 17]. Immature brain may be more amenable than the mature brain to their functional incorporation [18]. The patient in this paper is less than 10 years old, so she may benefit more from stem cell transplantation than adult patients.

There is increasing evidence showing that administration of MSCs may promote recovery in animal nervous disease models including ataxic and ischemic stroke, resulting from the secretion of particular neurotrophic factors [19, 20]. Also, MSCs have the advantage of being able to multiply and differentiate into neuronal or neuronal-like cells in the brain [21]. It is reported that many MSCs lodge in the lung with systemic delivery in an animal model and secrete anti-inflammatory factors [22]. The MSCs are implicated in immune regulation resulting from suppressing initial immune responses and clean up inflammatory factors from preexistence of immune responses [23]. Therefore, existence of both MSCs-specific cytokines as early effectors and the differentiated tissue-specific cytokines as later effectors could support brain cell and neuronal process repair. Here we also observed that the natural resistance to disease of the patient was strengthened; for example, she had less frequency of influenza after MSCs treatment.

In conclusion, umbilical cord MSCs transplantation showed the potential promise of, at least partially, improving the gross motor dysfunction of children with cerebral palsy. The result suggests that MSCs transplantation may be a safe and effective way to treat cerebral palsy. Efficacy and adverse effects in long term in a large-size cohort merit further investigation.

## Figures and Tables

**Table 1:** Transplantation details.

Date (year/month/day)	Total cell count	Cell viability
2008/12/3	6.3 × 10^6^	96.2%
2009/1/5	7.5 × 10^6^	95.1%
2009/2/6	10.2 × 10^6^	96.7%
2009/3/8	9.1 × 10^6^	96.3%
2009/4/7	5.7 × 10^6^	97.1%
2009/5/4	6.2 × 10^6^	95.6%
2009/6/9	8.5 × 10^6^	96.3%

**Table 2:** Comparison of functional independence measures before and after the last umbilical cord MSCs administration.

Items	Onset	Followed-up	
		3rd month	28th month
Self-care			
(1) Eating	3	4	6
(2) Grooming	2	3	4
(3) Bathing	2	3	4
(4) Dressing upper	3	4	7
(5) Dressing lower	3	4	7
(6) Toileting	3	4	7
(7) Bladder management	4	5	7
(8) Bowel management	4	5	7

Self-care total	24	32	49

Mobility			
(9) Transfers: chair/wheelchair	4	6	7
(10) Transfers: toilet	4	5	7
(11) Transfers: tub/shower	2	3	4
(12) Locomotion: walk/wheelchair/crawl	4	5	7
(13) Locomotion: stairs	3	4	7

Mobility total	17	23	32

Communication			
(14) Comprehension	3	4	5
(15) Expression	1	2	3
Social cognition			
(16) Social interaction	1	2	3
(17) Problem solving	1	2	4
(18) Memory	1	2	5

Cognition total	7	12	20

Total of FIM	48	67	101
